# New cut-off values for ferritin and soluble transferrin receptor for the assessment of iron deficiency in children in a high infection pressure area

**DOI:** 10.1136/jcp.2009.066498

**Published:** 2009-11-20

**Authors:** K S Phiri, J C J Calis, A Siyasiya, I Bates, B Brabin, M Boele van Hensbroek

**Affiliations:** 1 Malawi–Liverpool–Wellcome Trust Clinical Research Programme, College of Medicine, Blantyre, Malawi; 2 Emma Children’s Hospital AMC, University of Amsterdam, the Netherlands; 3 Liverpool School of Tropical Medicine, Liverpool, UK

## Abstract

**Background:**

Due to the potential risk of iron supplementation in iron replete children, it is important to properly identify children who may require iron supplementation. However, assessment of the iron status has proven to be difficult, especially in children living in areas with high infection pressure (including malaria).

**Aims and Methods:**

Biochemical iron markers were compared to bone marrow iron findings in 381 Malawian children with severe anaemia.

**Results:**

Soluble transferrin receptor/log ferritin (TfR-F index), using a cut-off of 5.6, best predicted bone marrow iron stores deficiency (sensitivity 74%, specificity 73%, accuracy 73%). In order to improve the diagnostic accuracy of ferritin or sTfR as a stand-alone marker, the normal cut-off value needed to be increased by 810% and 83% respectively. Mean cell haemoglobin concentration (MCHC), using a cut-off of 32.1 g/dl, had a sensitivity of 67% and specificity of 64% for detecting iron stores deficiency.

**Conclusion:**

TfR-F index incorporated the high sensitivity of sTfR, a proxy for cellular iron need, and the high specificity of ferritin, a proxy for iron stores. In areas with a high infection pressure, the TfR-F index best predicted iron deficiency. However, in settings where diagnostic tests are limited, MCHC may be an acceptable alternative screening test.

The examination of stained aspirates of bone marrow for haemosiderin has been considered the “gold standard” as a method for evaluation of iron status.^1^ This technique is invasive and therefore not suitable for screening. There are currently several laboratory assays available for assessing the iron status in children. These are widely used in clinics and include ferritin, serum iron, serum transferrin, total iron binding capacity (TIBC) and mean cell volume (MCV). However, these iron markers are considerably altered by inflammation, which limits their applicability, especially in areas with a high infection pressure. Surprisingly, it is still unclear to what extent adjustment of the recommended cut-off values for these iron markers is required in order to improve their diagnostic efficiency. To date there are no studies which have validated these iron markers against the “gold standard” (bone marrow iron content) in children living in areas endemic for malaria and other common infective conditions. In areas with a limited infection pressure, soluble transferrin receptor (sTfR) has been shown to be a promising new tool for the diagnosis of deficiency of iron stores.^2^

Due to a recent finding in Tanzania of an increased mortality in iron replete children receiving iron supplementation, there is an urgent need to be able to target iron therapy and prophylaxis programmes on the children with proven iron deficiency.^3^ To be able to do this there is a need for non-invasive and sensitive tests that distinguish iron stores deficiency from functional iron deficiency which is associated with anaemia of inflammation. We have evaluated the diagnostic accuracy of various iron markers against bone marrow iron assessment in children residing in an area of high infection pressure.

## Methods

This study formed part of a large case–control study investigating the aetiology of severe anaemia among Malawian children described in detail elsewhere.^4^ In summary, the study was conducted between July 2002 and July 2004 in malaria endemic areas of Blantyre and Chikwawa. Children aged 6–59 months, presenting to hospital with severe anaemia (Hb <5 g/dl) were enrolled as cases. Written informed consent was obtained from the guardians of the children, and the study was approved by the ethics committees of the University of Malawi and the Liverpool School of Tropical Medicine, UK.

### Clinical assessment and management

Children had a detailed medical examination performed before collecting a sample of venous blood. Under local anaesthesia, a bone marrow aspiration was performed from the anterior or posterior iliac crest. All bone marrows were performed in the presence of the child’s guardian and she/he was allowed to withdraw consent at any time during the procedure. All children received a blood transfusion and were managed according to standard hospital protocols.^4^

### Laboratory methods

On recruitment, haematological indices (including haemoglobin (Hb), mean cell volume (MCV) and mean cell haemoglobin concentration (MCHC)) were determined using the HemoCue B-Haemoglobin (HemoCue AB, Ängelholm, Sweden) and a coulter counter analyser (Beckman Coulter, Durban, South Africa). Ferritin, serum iron and serum transferrin were also assayed (Roche Diagnostics, Switzerland). sTfR was measured using an enzyme immunoassay (Ramco Laboratories, Texas, USA). Blood was cultured for 5 and 56 days for the presence of routine pathogens and mycobacteria, respectively, using an automated BacT/Alert system (BioMerieux Industry, Missouri, USA).

### Definitions and cut-off values

Malaria was defined as presence of *Plasmodium falciparum* asexual parasites in blood. HIV testing was done using two rapid tests according to WHO guidelines and discordant results were resolved by PCR.^5^

Internationally accepted cut-off values for biochemical iron markers used in this analysis were as follows: ferritin <30 μg/l^6^; serum iron <3.6 μmol/l; serum transferrin >3.6 g/l; TIBC >72 μmol/l (laboratory reference values); transferrin saturation <16%^6^; sTfR >8.3 mg/l (test kit reference value); MCV <67 fl (<2 years old) and <73 fl (2–5 years old); MCHC <32 g/l.^6^ Transferrin-ferritin (TfR-F) index was defined as [sTfR ÷ log ferritin].^7^ A TfR-F index value of >5.6 (using sTfR of >8.3 mg/l and ferritin of <30 μg/l) was used to define deficiency of iron stores.^8^ C-reactive protein (CRP) of >10 mg/ml was defined as increased.^9^

Bone marrow aspirates were prepared and stained using a commercial kit and according to manufacturer guidelines (HematoGnost Fe, Darmstadt, Germany). Smears were graded for iron and defined as positive when the fragment iron was <2.^8^ Bone marrow iron assessment was used as the gold standard for diagnosis of iron stores deficiency.

Statistical analysis for sensitivity, specificity, positive predictive value (PPV), negative predictive value (NPV) and accuracy for individual iron markers was calculated using SPSS V.11.0. Iron markers having either a sensitivity or specificity <20% were classified as poor predictors of iron stores deficiency and were dropped from further analysis. For the remaining iron markers, receiver operating characteristics (ROC) curves for identifying the optimal cut-offs for best identifying iron stores deficiency were constructed, and the corresponding areas under the curve (AUC^ROC^) for all the markers were compared. A new cut-off for each iron marker which provided maximal sensitivity and specificity was determined from ROC curves.^10^ KSP analysed the data but all others had access to the primary dataset.

## Results

A total of 381 children were recruited with an average age of 1.7 years (SD 1.1); 46.7% (178/381) were male (table 1). Sixty per cent of severely anaemic children had malaria parasites in their blood; CRP was raised in 89%. Ferritin levels were increased, with a mean concentration of 729.2 (1528.1) μg/l (table 2). The proportions of children that were iron deficient ranged from 1% using TIBC to 97.5% using serum transferrin.

**Table 1:** Baseline characteristics of cases

Characteristic	Result
Recruited	n = 381
Age (months)*	20.4 (12.8) [381]
Hb (g/dl)*	3.6 (0.8) [381]
CRP (mg/ml)*	11.1 (8.5) [346]
Male	178/381 (46.7%)
Wasted†	52/330 (15.8%)
Stunted‡	176/331 (53.2%)
Malaria	226/380 (59.5%)
HIV	33/345 (9.6%)
Bacteraemia	52/259 (20.1%)

*Mean (SD) [total number].

†Z-score <−2 weight-for-height.

‡Z-score <−2 height-for-age.

CRP, C-reactive protein; Hb, haemoglobin.

**Table 2:** Mean iron marker and the proportion of children classified as iron stores deficient using internationally accepted cut-off values

Iron marker	Mean (SD)	Normal levels	Proportion iron deficient (%)
Ferritin (μg/l)	729.2 (1528.1)	30–300	12.7
sTfR (μg/ml)	17.4 (15.8)	<8.3	73.2
TfR-F index	12.9 (28.1)	<5.6	46.4
Serum iron (μmol/l)	16.0 (15.7)	3.6–27.0	19.8
Serum transferrin (g/l)	2.2 (0.7)	2.0–3.6	2.5
Transferrin saturation (%)	41.4 (39.7)	>16	37.4
TIBC (μmol/l)	41.5 (12.7)	<72	1.0
MCHC (g/dl)	32.9 (7.8)	32.0–36.8	43.3
MCV (fl)			
<2 years	106.3 (81.4)	67–91	12.1
⩾2 years	117.7 (88.1)	73–89	21.5

MCHC, mean cell haemoglobin concentration; MCV, mean cell volume; sTfR, soluble transferrin receptor; TfR-F index, transferrin-ferritin index; TIBC, total iron binding capacity.

Table 3 shows sensitivity and specificity of various iron markers. Poor markers that were dropped from further analysis included serum transferrin, TIBC and MCV, which had a sensitivity of 0%, 0% and 4% (<2 years) and 17% (⩾2 years), respectively. Ferritin, serum iron and transferrin saturation had a markedly lower sensitivity than specificity. Conversely, sTfR had a high sensitivity of 90% and low specificity of 37%. The accuracy of a marker was highest for ferritin (79%) and lowest for sTfR (49%).

**Table 3:** Sensitivity and specificity of iron markers to identify children with iron stores deficiency using internationally accepted cut-off values and bone marrow iron as the “gold standard”

	True		False		Sensitivity	Specificity	Accuracy
	Positives	Negatives	Positives	Negatives			
Ferritin	5	76	3	19	21	96	79
sTfR	35	52	87	4	90	37	49
TfR-F index	16	55	18	7	70	75	74
Serum iron	7	93	13	22	24	88	74
Serum transferrin	0	127	0	33	0	100	79
Transferrin Saturation	10	80	26	19	35	76	67
TIBC	0	127	0	33	0	100	79
MCHC	22	81	40	13	63	67	66
MCV							
<2 years	1	71	8	22	4	90	71
⩾2 years	2	36	7	10	17	84	69

AUC, area under the curve; MCHC, mean cell haemoglobin concentration; MCV, mean cell volume; ROC, receiver operating characteristics; sTfR, soluble transferrin receptor; TfR-F index, transferrin-ferritin index; TIBC, total iron binding capacity.

**Figure 1:**
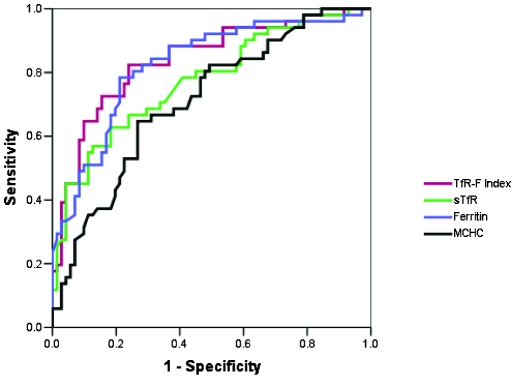
Received operating characteristic curves of soluble transferrin receptor (sTfR), ferritin and transferrin-ferritin index (TfR-F index) in the identification of iron stores deficiency. MCHC, mean cell haemoglobin concentration.

ROC curves for defining the optimal cut for the identification of deficiency of iron stores for ferritin, sTfR, TfR-F index, and other iron markers were constructed (fig 1). The area under the curve (AUC^ROC^) for each marker represented their performance (table 4). Ferritin, sTfR, TfR-F index and MCHC had significantly higher AUC^ROC^ than 0.5, where 0.5 signified an ineffective test. Serum iron and transferrin saturation were excluded from further analysis as they had non-significant AUC^ROC^ (0.62 and 0.60, respectively).

**Table 4:** AUC^ROC^ values for biochemical iron markers to identify children with deficiency of iron stores based on new cut-off values

	AUC^ROC^	SE	p Value
Ferritin	0.82	0.05	<0.001
sTfR	0.80	0.04	<0.001
TfR-F index	0.79	0.06	<0.001
Serum iron	0.58	0.06	0.2
Transferrin saturation	0.57	0.06	0.3
TIBC	0.52	0.06	0.8
MCHC	0.68	0.06	0.001

AUC, area under the curve; MCHC, mean cell haemoglobin concentration; ROC, receiver operating characteristics; sTfR, soluble transferrin receptor; TfR-F index, transferrin-ferritin index; TIBC, total iron binding capacity.

For the remaining iron markers, ferritin, sTfR, TfR-F index and MCHC, new cut-off levels, with an optimal combination of sensitivity and specificity, were determined from the ROC curves. Table 5 shows the resultant sensitivity and specificity, and the percentage change from the original cut-off. The ability of sTfR or ferritin to predict iron stores deficiency (accuracy) was similar and above 75% using the derived cut-offs of 273 μg/l and 15.2 μg/ml, respectively. Although the sensitivity and specificity for TfR-F index or MCHC (sensitivity 74%, 67%; and specificity 73%, 64%, respectively) were lower than those for ferritin and sTfR, they required smaller changes from their original to new cut-off levels.

**Table 5:** Ability of ferritin, sTfR, TfR-F index and MCHC to identify children with deficiency of iron stores based on new cut-off values

	Original cut-off	New cut-off	% change in cut-off	Sensitivity*	Specificity*	Accuracy*
Ferritin	30 μg/l	273 μg/l	810	75	76	76
sTfR	8.3 μg/ml	15.2 μg/ml	83	77	76	76
TfR-F index	5.6	5.3	−5	74	73	73
MCHC	32.0 g/l	32.1 g/l	0.3	67	64	65

*Sensitivity, specificity or accuracy of new cut-off.

MCHC, mean cell haemoglobin concentration; sTfR, soluble transferrin receptor; TfR-F index, transferrin-ferritin index.

## Discussion

In the present study, the diagnostic efficiency of sTfR and a variety of more conventional laboratory tests for the identification of deficiency of iron stores or functional deficiency was evaluated. The results suggested that serum transferrin, TIBC and transferrin saturation were of limited value in diagnosis of deficiency of iron stores as their corresponding AUC^ROC^ values did not provide acceptable sensitivity and specificity estimates. Most likely, this relates to the interference of inflammatory cytokines produced as part of the acute phase response during an infection.^11^ ^12^

The bone marrow iron smear was used as the “gold standard” for the diagnosis of deficiency of iron stores. It has generally been considered the most reliable diagnostic test, but has the limitations of being more invasive than peripheral blood iron markers.^1^ Furthermore, incorrect assessment of iron stores in bone marrow aspirates has been described.^13^

Ferritin has been widely used as an iron marker in individuals without inflammatory conditions.^6^ Conversely, sTfR is considered to reflect the degree of tissue iron need, and there is evidence that it is a good indicator of iron status when the iron stores are depleted.^14^ The reciprocal relationship between sTfR and ferritin describes a perfect log-linear relationship over a wide range of normal and depleted iron stores states. Punnonen *et al*^7^ evaluated various possibilities of combinations of sTfR and ferritin parameters, and concluded that use of the sTfR/log ferritin ratio (TfR-F index) considerably improved diagnostic efficiency, even in settings with a high infection pressure.

In the present study, serum ferritin had a high specificity and sTfR a high sensitivity, which is consistent with previous studies.^15^ Changing the current conventional cut-off of TfR-F index (5.6) to its optimal cut-off (according to the ROC analysis) of 5.3 has little effect on its diagnostic efficiency. This is in contrast with ferritin and sTfR which required a change of 810% and 80%, respectively, to achieve maximal sensitivity and specificity.

During a study in which healthy volunteers were serially phlebotomised to induce iron deficiency, MCHC was found to be a sensitive early indicator of iron deficient erythropoiesis.^16^ ^17^ In the present study, MCHC had a relatively good diagnostic efficiency. This is relatively good news since MCHC can be measured using a coulter counter, which is relatively cheap and more widely available in resource-limited settings as compared with either ferritin or sTfR.

Results from the present study suggest that it is necessary to change the cut-off limit for ferritin from 30 to 273 μg/l in order to improve its diagnostic efficiency. This proposed increase is consistent with other studies and probably reflects the effect of the acute phase response on ferritin levels.^7^ Witte *et al*^18^ developed a nomogram describing the relationship between ferritin and CRP or erythrocyte sedimentation rate, to detect or exclude iron deficiency in patients with anaemia of inflammation in order to minimise bone marrow examination. Unfortunately, when this nomogram, which corrected for the acute phase component of changes in ferritin, was applied in later studies, it performed poorly.^19^

Results from the present study indicates that ferritin and sTfR are relatively good markers for detecting iron stores deficiency provided that new cut-off values are applied. Combining these markers into the TfR-F index may prove to be a much better tool to detect iron deficiency in children in these high infection pressure areas. The cut-off values for the TfR-F index are robust and do not need to be adjusted for inflammation.

Our study is among the largest and most detailed investigations into assessment of the iron status in children in this setting and has generated a unique dataset. However, the fact that we did not do a healthy population-based study, but focused on a subgroup of severely anaemic children, may limit generalisability of our findings, especially in view of the fact that these children also tend to have other inflammatory conditions that may affect interpretation of ferritin levels. Obvious ethical issues, of performing bone marrow investigations in mild and non-anaemic children, was the main reason for focusing on this study population. However, these findings may be a starting point and may provide an improved knowledge of diagnostic criteria for iron status assessment that avoids the need to do a bone marrow aspiration, and be of value for determining therapeutic practice. This is especially important taken the recent observations that iron supplementation to iron replete children may be fatal.^3^

**Take-home messages**

- There is a need to properly diagnose true iron deficiency due to the probable increased risk of adverse effects associated with iron supplementation in iron replete individuals.
- Transferrin-ferritin index is probably the most useful and robust iron marker that best predicts bone marrow iron status.
- Mean cell haemoglobin concentration may be an acceptable alternative screening test in resource-poor settings.
